# The impact of neurological performance and volumetrics on overall survival in brain metastasis in colorectal cancer: a retrospective single-center case series

**DOI:** 10.1186/s12885-022-09435-1

**Published:** 2022-03-28

**Authors:** Caroline Sander, Clara Frydrychowicz, Gordian Prasse, Sabine Taubenheim, Felix Arlt, Jürgen Meixensberger, Michael Karl Fehrenbach

**Affiliations:** 1 Department of Neurosurgery, University Hospital Leipzig, Liebigstrasse 20, 04103 Leipzig, Germany; 2 Division of Neuropathology, University Hospital Leipzig, Liebigstrasse 26, 04103 Leipzig, Germany; 3 Institute of Neuroradiology, University Hospital Leipzig, Liebigstrasse 20, 04103 Leipzig, Germany; 4 Leipzig Cancer Registry, University Hospital Leipzig, Philipp-Rosenthal-Strasse 27b, 04103 Leipzig, Germany

**Keywords:** Colorectal cancer, Brain metastasis, Neurologic performance, Prognostic factors, Radiotherapy, Volumetrics

## Abstract

**Background:**

Brain metastasis (BM) of colorectal cancer is a disease with a poor prognosis of only a few months survival. However, it is difficult to estimate the individual prognosis of each patient due to the lack of definitive prognosis parameters. The number of metastases and the Karnofsky performance score are known predictors for survival. We investigated whether or not the neurological performance score and the tumor volumetrics are equally suitable predictors for survival.

**Design:**

All patients with histologically diagnosed BM linked to colorectal cancer between 2012 and March 2020 were reviewed. The Medical Research Council Neurological Performance Score was used to quantify neurological performance. Univariate analysis with Kaplan-Meier estimate and log-rank test was performed. Survival prediction and multivariate analysis were performed employing Cox proportional hazard regression.

**Results:**

Twenty-five patients were included in our analysis with an overall survival of 4.9 months after surgery of the BM. Survival decreased in the univariate analysis with increasing postoperative neurological performance score, low Karnofsky performance score, absence of radiation therapy and radiation therapy modality. The neurological performance score is a reliable scoring parameter for estimating the prognostic course analogous to the Karnofsky performance score. Neither preoperative nor post resection residual tumor volume had any impact on overall survival in our small cohort.

**Conclusion:**

Our data suggest that the postoperative neurological performance is a valuable prognostic factor for colorectal cancer patients with BM. Tumor volumetrics show no correlation to survival. Further investigations with a larger number of cases are mandatory.

**Supplementary Information:**

The online version contains supplementary material available at 10.1186/s12885-022-09435-1.

## Introduction

The incidence of brain metastases (BMs) in colorectal cancer (CRC) is very rare and is estimated to be between 0.6 and 3.2% [1–4]. The BMs occur at late stages of the disease with a median from first diagnosis of CRC to BM of over two years [5, 6]. They can, therefore, be considered as a result of increased survival, improved treatment options of the underlying disease and better detection of even small metastases by magnetic resonance imaging [4, 7]. The development of BM is metachronic and followed up from 24 [8] to 44.6 months [9], depending on the stage of the initial manifestation of the CRC [10]. Nevertheless, the development of the BM is associated with a very poor prognosis and varies between 2 and 8 months after the diagnosis of BM [4–6, 8–15].

The survival rates for one and two years are very low at 21.1 and 3.3%, respectively [10]. In addition to the poor prognosis of patients with BM, they often complain of neurological impairments, such as headaches, dizziness, speech disorders or hemiparesis, due to the localization of the metastases in eloquent brain areas. The reduced neurological condition of the patients often results in a poor quality of life [16]. There are no promising treatment options apart from aggressive surgery followed by radiotherapy [17]. Studies on gamma knife surgery have shown no survival benefits compared to tumor resection followed by radiotherapy [18]. A targeted therapy is essential to improve the overall prognosis of patients.

There is a lack of prognostic markers in the literature on the estimation of patients with BM. An exact prognostic assessment of the patients is essential and helpful for decision-making regarding further therapy measures. There are considerable differences in everyday clinical practice between patients concerning overall survival [16]. A prognostic score for the synchronous metastasis of CRC and BM was established recently in a comprehensive study [16]. The number of metastases and the Karnofsky performance score (KPS) were identified as relevant prognostic factors.

A positive prognostic value in patients with glioblastoma, a malignant brain tumor, was recently assigned to the extent of neurological damage postoperatively compared to preoperatively [19]. The authors also describe a strong connection between residual tumor volume postoperatively and the prognosis.

The literature lacks studies on patients with CRC and the influence of metastasis volumes, the extent of resection and the Medical Research Council Neurological Performance Scale (MRC-NPS) on overall survival. The aim of this study was to analyze the impact of these parameters in CRC patients with BM.

## Material and methods

Data collection and analysis were approved by the ethical committee of the Medical Faculty, University of Leipzig (No. 005/17-ek), and carried out in accordance with data protection guidelines. Informed consent for retrospective data analysis was obtained from all patients treated in the Neurosurgical Department of Leipzig University. The study included patients with BM from CRC between 2012 and March 2020. The date of neurosurgery was chosen as date of diagnosis of BM. The following variables were collected: Age, gender, primary tumor site, tumor grade (G1, G2, G3), *Union for International Cancer Control* (UICC) state (UICC I, II, III, IV) according to the international classification system [20], neoadjuvant or adjuvant therapy, BM localization and the modality of CNS radiation therapy (stereotactic or whole brain). Data for liver, lung and other metastases were obtained from clinical records or outpatient service records. Overall survival was defined as the time span between the date of neurosurgery and date of death. The date of death, if not provided by our hospital database, was collected from the Leipzig Cancer Registry.

The neurological performance status and Karnofsky performance status were routinely assessed at the time of hospital admission (preoperative) and within 24 h after cranial surgery (postoperative). The 5-point MRC-NPS adjusted by Bleeheen et al. [21] was used to quantify the severity of neurological deficits with (1) no neurological deficit, (2) some neurological deficit but function adequate for useful work, (3) neurological deficit causing moderate functional impairment (difficulty to move limbs, moderate dysphasia, moderate paresis, some visual disturbance), (4) neurological deficit causing major functional impairment (inability to move limbs, gross speech or visual disturbances) and (5) inability to make conscious responses.

We measured the tumor volume by manual segmentation in BRAINLAB (iPlan Net, version 3.0.5 Cranial, Brainlab AG, Munich, Germany) using magnetic resonance imaging scans (gadolinium-enhanced sequence t1). The tumor load was defined as the sum of all BM volumes per patient. We further distinguished between pre- and postoperative tumor load and infratentorial and supratentorial tumor load.

### Statistical analysis

The statistical analysis was carried out using SPSS statistics software version 25.0.0 (IBM, Armonk, New York State, USA) and Prism version 8.2.0 (GraphPad Software, Inc., San Diego, USA). Overall survival was calculated using the Kaplan–Meier function. If death did not yet occur or if patients were lost to follow-up, the date of last contact was included in the statistical analysis as a censored value.

The significance between survival curves were tested by log-rank test. Survival rates were given for three (median survival) and five months (mean survival) with standard deviation. A survival prediction model for the impact of clinicopathological variables was calculated with Cox regression. Metric variables were categorized using the mean. All factors with a statistical significance below 0.05 from univariate analysis were utilized for a multivariate analysis via proportional hazard Cox regression in order to investigate independent prognostic factors for overall survival. The metric variables were categorized into two groups according to the median. Hazard ratios are provided with 95% confidence interval (CI) and were considered statistically significant if 1 was excluded by 95% CI.

A t-test was used to identify a statistical difference between metric variables; the exact Fisher test was used for the categorical variables. *P*-values below 0.05 were considered statistically significant.

## Results

A total of 25 patients were enrolled in the study over a period of eight years (see Table 1 for patient characteristics). One patient had a second symptomatic BM during this period, which led to a second surgery after six months. The median age of the patient at first diagnosis of CRC was 63 years (range 30 – 81 years). The most frequent grading at the time of first diagnosis was G3 (36%), and the majority of patients were at UICC stage III. Only nine patients (36%) had metastases at the time of the initial diagnosis of CRC (three patients with synchronous lung metastases (12%) and five cases with synchronous liver metastases (20%). The symptomatic BM led to the diagnosis of a CRC in two patients.

**Table 1:** Demographics of patients with BM

		total CRC *N* = 25		colon cancer *N* = 11		rectal cancer *N* = 14	
		Number (%)	mean (min-max)	Number (%)	mean (min-max)	Number (%)	mean (min-max)
**patient characteristics**							
gender, female		6 (24.00)		5 (45.45)		1 (7.14)	
age at first diagnosis, yr			61.88 (30–81)		59.91 (30–81)		63.43 (51–78)
age at first diagnosis, > 63 yr		10 (40.00)		4 (36.36)		6 (42.86)	
body mass index, kg/cm^2^			26.1 (19.1–41.6)		26.3 (19.1–41.6)		25.9 (22.0–32.4)
high blood pressure		13 (52.00)		7 (63.64)		6 (42.86)	
alcohol consumption		2 (8.00)		0		2 (14.29)	
smoking history		2 (8.00)		1 (9.09)		1 (7.14)	
convulsion		6 (24.00)		1 (9.09)		5 (35.71)	
cardiovascular disease		7 (28.00)		4 (36.36)		3 (21.43)	
diabetes mellitus type II		4 (16.00)		4 (36.36)		0	
second malignoma		2 (8.00)		1 (9.09)		1 (7.14)	
chronic kidney injury		4 (16.00)		2 (18.18)		2 (14.29)	
chronic obstructive pulmonic disease		4 (16.00)		2 (18.18)		2 (14.29)	
cardiac stents		1 (4.00)		0		1 (7.14)	
adipositas		4 (16.00)		2 (18.18)		2 (14.29)	
**colorectal cancer**							
UICC stage	n/a	2 (8.00)		0		2 (14.29)	
	I	3 (12.00)		1 (9.09)		2 (14.29)	
	II	1 (4.00)		1 (9.09)		0	
	III	10 (40.00)		6 (54.55)		4 (28.57)	
	IV	9 (36.00)		3 (27.27)		6 (42.86)	
Grading	n/a	2 (8.00)		1 (9.09)		1 (7.14)	
	G1	9 (36.00)		6 (54.55)		3 (21.43)	
	G2	8 (32.00)		3 (27.27)		5 (35.71)	
	G3	6 (24.00)		1 (9.09)		5 (35.71)	
pulmonary metastasis		18 (72.00)		7 (63.64)		11 (78.57)	
time from first diagnosis to pulmonary metastasis, mo			33.44 (0–103)		37.43 (0–82)		30.91 (0–703)
liver metastasis		15 (60.00)		8 (72.73)		7 (50.00)	
time from first diagnosis to liver metastasis, mo			19.27 (0–98)		30.29 (0–98)		9.63 (0–40)
other systemic metastasis (without brain)		9 (36.00)		3 (27.27)		6 (42.86)	
number chemotherapeutic drugs			3.08 (0–8)		3.64 (0–8)		2.64 (0–7)
systemic chemotherapy	w/o	2 (8.00)		0		2 (14.29)	
	adjuvant	14 (56.00)		11 (100.00)		3 (21.43)	
	neoadjuvant + adjuvant	9 (36.00)		0		9 (64.29)	
systemic radiation therapy	w/o	14 (56.00)		8 (72.73)		6 (42.86)	
	neoadjuvant	4 (16.00)		0		4 (28.57)	
	adjuvant	7 (28.00)		3 (27.27)		4 (28.57)	
**brain metastasis**							
age at diagnosis of BM, yr			65.80 (36–82)		64.27 (36–82)		67.00 (51–78)
time from first diagnosis to BM, mo			47.16 (0–102)		52.91 (5–95)		42.64 (0–102)
number of BMs	1	14 (56.00)		9 (81.82)		5 (35.71)	
	2	3 (12.00)		0		3 (21.43)	
	3	6 (24.00)		1 (9.09)		5 (35.71)	
	> 3	2 (8.00)		1 (9.09)		1 (7.14)	
localization of BM	cerebral	9 (36.00)		6 (54.55)		3 (21.43)	
	cerebellar	6 (24.00)		3 (27.27)		3 (21.43)	
	both	10 (40.00)		2 (18.18)		8 (57.14)	
symptoms of BM	incidental finding	4 (16.00)		3 (27.27)		1 (7.14)	
	unspecific CNS	3 (12.00)		3 (27.27)		0	
	specific CNS	18 (72.00)		5 (45.45)		13 (92.86)	
radiation therapy, brain		17 (68.00)		7 (63.64)		10 (71.43)	
radiation therapy, modality	stereotactic	11 (64.71)		5 (71.43)		6 (60.00)	
	whole brain	6 (35.29)		2 (28.57)		4 (40.00)	
radiation dose, Gy			38.31 (25–71)		33.50 (25–45)		41.20 (30–71)
**surgery**							
localization surgery	cerebral	11 (45.83)		6 (54.55)		5 (38.46)	
	cerebellar	13 (54.17)		5 (45.45)		8 (61.54)	
cross total resection		13 (54.17)		8 (72.73)		5 (38.46)	
age at death, yr			66.20 (37–81)		62.56 (37–81)		69.18 (51–79)
time from first diagnosis to death, mo			51.75 (5–104)		56.44 (9–704)		47.91 (5–95)
time from BM to death, mo			4.90 (0–18)		6 (0–18)		4 (0–9)
time from BM to death, mo [median]			3.00 (0–18)		3 (0–18)		2 (0–9)
six-month survival, mo		8 (40.00)		3 (33.33)		5 (45.45)	
one-year survival, mo		2 (10.00)		2 (22.22)		0	

*BM* brain metastasis, *CI* confidence interval, *CRC* colorectal cancer, *CNS* central nervous system, *G* grading, *Gy* Gray, *HR* Hazard ratio, *KPS* Karnofsky performance status, *Mo* months, *MRC-NPS* Medical Research Council Neurological Performance Ccore, *N* number, *n/a* not applicable, *OS* Overall survival, *UICC* Union for International Cancer Control, *Yr* years, *w/o* without

The majority of patients had localization-specific symptoms (18 patients; 72%) which led to the diagnosis of BM; three patients (12%) suffered from unspecific cranial symptoms like headache or nausea, the BM was an incidental finding for four patients (16%). In addition, 36% of patients (9 cases) developed following to the BM further systemic metastasis of the bones (60%), peritoneum (20%), spleen (10%) or mediastinum (10%).

Nearly all patients (92%) received systemic therapy after the diagnosis of CRC. Patients received and average of three different chemotherapeutic agents or immunomodulators (median: 3, range: 0 – 8) before diagnosis of BM (used chemotherapeutics: 5-fluoruracil, leucovorin, irinotecan, oxaliplatin, trifluridine/ tipiracil, capecitabin; used immunomodulators: bevacizumab, cetuximab, panitumumab, ramucirumab, aflibercept, regorafenib). Four patients received radiotherapy of the rectum (16%) and seven patients were irradiated at distant metastases (28%, excluding BMs).

The BMs developed an average of 47.2 months (median: 51 months, range: 0 – 102 months) after the initial diagnosis of CRC. A singular BM was present in 13 cases (52%; systemic metastases: lung metastasis: 72% of all patients, liver metastasis: 60% of all patients, spleen metastasis: 4% of all patients, peritoneal metastasis: 8% of all patients, bone metastasis: 20% of all patients, mediastinal metastasis: 4% of all patients) and a solitary metastasis in three cases (12%). The BM was located primarily in the cerebrum (9 cases, 36%), cerebellum (six cases, 24%) and both compartiments (10 cases, 40%). If there was multifocal manifestation, the symptomatic metastasis was removed (cerebellum 54.2%). In one case resections of occipital and cerebellar metastases were performed.

### Overall survival

Patients died 51.7 months (median: 51, range: 5 – 104 months) after first diagnosis and 4.9 months (median: 3, range: 0 – 18 months) after BM diagnosis. The mean age of death was 66 years (median 66 yr, range 37 – 81 yr). Survival rates three and five months after diagnosis of brain lesions were estimated by Kaplan Meier survival curves and significance was tested by log-rank test (Table S1). Two patients showed long-term survival of 14 and 18 months, both were male, had UICC stage III at first diagnosis and developed systemic metastases in the lung and liver. P1 was 30 years old at initial diagnosis and 36 years old at BM detection; P2 was 57 years old and 63 years old at BM diagnosis. Both showed a singular BM and received stereotactic radiation of the lesion over the course of the disease.

### Volumetrics

A little more than half of the patients (13 cases, 52%) had a singular/solitary BM.

Patients had an average of 1.6 metastases (median: 1, range: 1 – 6), most of them in the cerebrum and cerebellum (10 cases, 40%). Patients with rectal carcinoma had the most BM in the cerebral and cerebellar (8 cases, 57.14%), patients with colon carcinoma had the most BM in the cerebrum (6 cases, 54.54%; Table 1). The BM localization was independent of the primary localization of the CRC. The average preoperative tumor load was 15.6 cm^3^ (median: 14.6 cm^3^, range 1.73 – 29.46 cm^3^) and the average postoperative tumor load was 3.38 cm^3^ (median: 0 cm^3^, range 0 – 19 cm^3^). The extent of tumor resection was 12.53 cm^3^ (median: 11.2 cm^3^, range 1.73 – 28.36 cm^3^). A complete resection was possible in 13 cases (52%), of which two patients (8%) had multiple metastases (Table 2).

**Table 2:** Perioperative volumetrics of brain metastases

	total	colon cancer	rectal cancer
	*N* = 25	*N* = 11	*N* = 14
	mean (min–max)	mean (min–max)	mean (min–max)
tumor volume of operated BM, cm^3^	12.63 (1.73–28.66)	13.27 (1.73–25.99)	12.13 (4.70–28.66)
preoperative tumor load, cm^3^	15.90 (1.73–29.46)	15.07 (1.73–25.99)	16.56 (4.70–29.46)
postoperative tumor load, cm^3^	3.38 (0–19.00)	2.05 (0–10.95)	4.42 (0–19.00)
difference tumor loads pre−/postoperative, cm^3^	12.53 (1.73–28.36)	13.02 (1.73–25.99)	12.14 (4.70–28.36)
tumor volume cerebellar, preoperative, cm^3^	8.25 (0–28.66)	8.45 (0–25.99)	8.08 (0–28.66)
tumor volume cerebellar, postoperative, cm^3^	0.53 (0–7.49)	0.79 (0–7.49)	0.32 (0–3.30)
*BM* brain metastasis, *N* number			

### Radiation therapy

After resection of the metastasis, 70.8% of patients (17 cases) received adjuvant brain radiotherapy in the form of stereotactic (11 patients, 44%) or whole brain radiation (6 patients, 24%). Most of the patients who were not irradiated were classified as non-irradiatable by the KPS (KPS < 70%: 4 cases (66.67%), *p*-value 0.038, exact Fisher test) or MRC-NPS (MRC-NPS ≥ 4: 4 cases (66.67%), *p*-value 0.038, exact Fisher test); irradiation was declined by the patients in a few cases (3 cases, 12%). The patients with whole brain radiation had a mean of 2.5 metastases (median 2, range 1 – 6) and a total tumor load of 20.95 cm^3^ (median: 21.62 cm^3^, range: 9.76 – 29.56 cm^3^). By comparison, patients with stereotactic radiation had an average of 1.5 metastases (median: 1, range 1 – 3, *p*-value 0.175, t-test) and a preoperative tumor load of 13.18 cm^3^ (median: 10.76 cm^3^, range: 2.92 – 26.35 cm^3^, *p*-value: 0.082, t-test). Postoperatively, patients with whole brain radiation had a tumor load of 3.42 cm^3^ (median: 0.36 cm^3^, range: 0 – 10.41 cm^3^), while patients with stereotactic radiation had an average of 3.96 cm^3^ (median: 0 cm^3^, range: 0 – 19 cm^3^).

### Neurological performance

Patients were preoperatively in a good neurological (72% MRC-NSP: 1 or 2) and clinical status (KPS: 100% ≥ 70 KPS, Table 3). Seven patients experienced a worsening of the neurological status postoperatively (preoperative MRC-NPS difference: mean: 0.64, median: 0, range 0 – 3) and 10 patients a worsening of the KPS (preoperative KPS difference: mean: 16, median 10, range: 0 – 70). One patient died within a few days of surgery from a postoperative complication with intracerebral hematoma. One patient had not received surgery due to a rapid deterioration of his condition resulting in early death.

**Table 3:** Clinical and neurological performance scores perioperative

		total CRC	colon cancer	rectal cancer
		*N* = 25	*N* = 11	*N* = 14
		Number (%)	Number (%)	Number (%)
preoperative KPS	100	2 (8.00)	0	2 (14.29)
	90	9 (36.00)	5 (45.45)	4 (28.57)
	80	7 (28.00)	4 (36.36)	3 (21.43)
	70	7 (28.00)	2 (18.18)	5 (35.71)
postoperative KPS	100	1 (4.00)	0	1 (7.14)
	90	4 (16.00)	2 (18.18)	2 (14.29)
	80	4 (16.00)	2 (18.18)	2 (14.29)
	70	10 (40.00)	4 (36.36)	6 (42.86)
	50	2 (8.00)	2 (18.18)	0
	40	1 (4.00)	0	1 (7.14)
	20	2 (8.00)	0	2 (14.29)
	10	1 (4.00)	1 (9.09)	0
KPS difference	0	10 (40.00)	3 (27.27)	7 (50.00)
	10	6 (24.00)	4 (36.36)	2 (14.29)
	20	5 (20.00)	3 (27.27)	2 (14.29)
	50	2 (8.00)	0	2 (14.29)
	60	1 (4.00)	0	1 (7.14)
	70	1 (4.00)	1 (9.09)	0
preoperative MRC-NPS	1	9 (36.00)	7 (63.64)	2 (14.29)
	2	9 (36.00)	2 (18.18)	7 (50.00)
	3	7 (28.00)	2 (18.18)	5 (35.71)
	4	0	0	0
	5	0	0	0
postoperative MRC-NPS	1	8 (32.00)	6 (54.55)	2 (14.29)
	2	6 (24.00)	2 (18.18)	4 (28.57)
	3	5 (20.00)	0	5 (35.71)
	4	3 (12.00)	2 (18.18)	1 (7.14)
	5	3 (12.00)	1 (9.09)	2 (14.29)
MRC-NPS difference	0	18 (72.00)	8 (72.73)	10 (71.43)
	1	1 (4.00)	0	1 (7.14)
	2	3 (12.00)	1 (9.09)	2 (14.29)
	3	3 (12.00)	2 (18.18)	1 (7.14)

CRC colorectal cancer,* KPS* Karnofsky performance status, *MRC-NPS* Medical Research Council Neurological Performance Score, *N* number

### Prognostic factors

No influence of the different pre- and postoperative tumor volumes could be identified in the investigation of individual factors on survival. However, there was a statistically significant difference in survival related to the performance of brain radiation (*p*-value: 0.0078, log-rank test) and the different brain radiation modalities (*p*-value: 0.009, log-rank test). A good neurological postoperative condition of the patient yielded a survival benefit (MRC-NPS ≥ 4; *p*-value: 0.006, log-rank test; see Table S1). It is worth noting that all patients had a good neurological status preoperatively; the MRC-NPS did not exceed 3. Postoperatively, on the day of discharge, the MRC-NPS was an average of 2, 0.64 points worse than preoperatively. Survival decreased with increasing postoperative MRC-NPS (*p*-value 0.001, log-rank test) and MRC-NPS difference (*p*-value 0.006, log-rank test). The KPS behaves equivalently. The preoperative MRC-NPS and KPS had no influence on survival. Subgroups were employed to assess the influence of neurological performance and radiotherapy more accurately. Patients who received radiotherapy and were of good neurological status (MRC-NPS < 4) had a significantly better survival (log-rank *p*-value 0.003, 3-month survival 70.1% (± 12.6%) compared to the other groups (without radio/MRC-NPS ≥ 4: 3-month survival of 33.33%, ± 27.2%; without radio/MRC-NPS < 4 and radio/ MRC- NPS ≥ 4: 3-month survival of 0% each).

Other factors were examined and showed no significant effect on the overall survival (See Table S1).

**Fig. 1:**
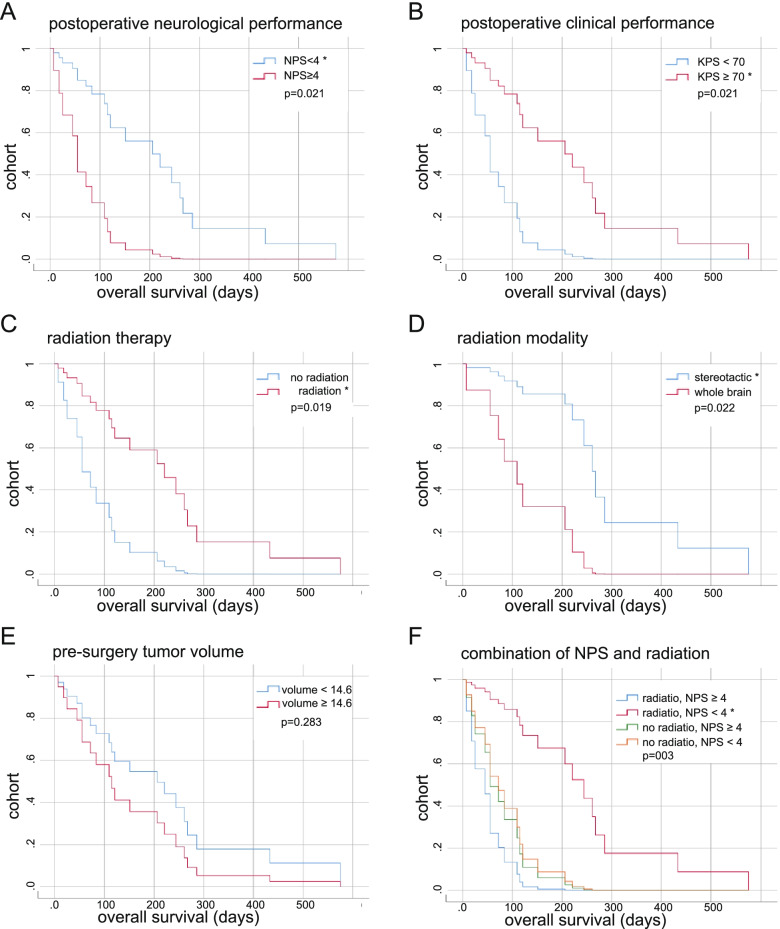
Predicted overall survival in CRC. Survival time analyzed for postoperative MPC-NPS divided into less than 4 and greater or equal to 4 (**A**); survival time depending on postoperative KPS less than 70 and greater or equal to 70 (**B**); survival time depending on radiation therapy (**C**) and radiation modality (**D**); pre-surgery tumor volume (**E**) and analysis of four subgroups and plotted survival time depending on radiation therapy and NPS (**F**). All diagrams via COX regression. * indicates significant *p*-values (COX Regression)

We used Cox proportional hazard regression to identify prognostic factors for survival. Good neurologic (MRC-NPS ≥ 4), good clinical status (KPS ≥ 70) postoperatively, performance of brain irradiation and irradiation modality were identified as prognostic factors on prolonged survival at the univariate level (Table 4, Fig. 1). Tumor volumes had no predictive effect on survival (Fig. 1), neither did other preclinical and clinical factors (Table S2). However, multivariate testing did not identify any of these factors as independently relevant regarding overall survival (Table 4).

**Table 4:** Univariate and multivariate survival analysis performed by Cox proportional hazard regression with factors from univariate analysis *p* >  0.05. The metric variables were dichotomized according to the median

	HR (95% CI)	*p*-value
univariate		
postoperative KPS, ≥ 70	4.25 (1.25–14.45)	0.021
postoperative MRC-NPS, ≥ 4	4.25 (1.25–14.45)	0.021
radiation therapy brain	0.25 (0.08–0.80)	0.019
radiation therapy modality, whole brain	5.40 (1.27–22.88)	0.022
preoperative tumor load, > 14.59 cm^3^	1.66 (0.66–4.19)	0.283
postoperative tumor load, > 0 cm^3^	1.03 (0.41–2.60)	0.948
multivariate		
postoperative KPS, ≥ 70	0.16 (0.02–1.18)	0.072
postoperative MRC-NPS, ≥ 4	a	
radiation therapy brain	a	
radiation therapy modality, whole brain	4.36 (0.95–20.02)	0.059

*HR* Hazard ratio,* KPS* Karnofsky performance status, *MRC-NPS* Medical Research Council Neurological Performance Score. *a* degree of freedom reduced due to constant or linear dependent covariates, constant or linearly dependent covariates postoperative MRC-NPS ≥ 4, postoperative KPS ≥ 70

## Discussion

The characteristics of our collective and their survival times are comparable to previously published data from other authors [4, 6, 8, 14]. A precise understanding of prognostic factors is relevant for therapy planning due to the heterogeneity of the tumor disease and the vastly different survival rates for patients after the manifestation of BM. We demonstrated that the postoperative neurological state and the difference between pre- and postoperative exerts an influence on the overall survival on a univariate level. The postoperative MRC-NPS is suitable to discriminate between long and short survival after tumor surgery. The data from our collective of patients with BM in CRC confirms the results of a prognostic estimation by the neurological performance score from brain tumors [19]. The MRC-NPS is based on the KPS, with the same outcome parameters identified. Our results regarding KPS as a prognostic factor is consistent with previous publications on patients with CRC [7], but also other tumor diseases, such as gastric cancer, esophageal cancer, non-small cell lung cancer, breast cancer, renal cell cancer, and malignant melanoma [7, 22] or glioblastoma [19].

Other prognostic factors we analyzed were radiotherapy and the modality of brain radiation. In line with recent studies, we found that radiotherapy is an important part of tumor therapy and prolongs the survival of patients with CRC and BM [9, 12, 17, 23]. It is noteworthy that only patients with an MRC-NPS < 4 benefited from radiation. The neurological status was independent of the radiation in the estimation of survival but only for the good conditions (MRC-NPS < 4). This shows that poor neurological status has a greater impact on overall survival than adjuvant radiotherapy.

The modality of radiation therapy also influenced the overall survival. Patients with whole brain irradiation survived for significantly less time than those with stereotactic radiation. We suspect that the patients with whole brain radiation therapy had a higher tumor burden and were concordant to the known prognostic number of BM with a poor outcome [15, 23].

The number of BM and the tumor volume did not have a statistically significant influence on the radiation modality, but a trend was observed. The preoperative, postoperative and infratentorial volumes of BMs had no influence on survival. This also includes the extent of resection measured in cm^3^. These findings are in contrast to reports from other authors [10, 15, 17]. The lack of statistical significance in our patients is most probably caused by the relative small group size.

Factors such as age [1, 16, 17], type and extent of history of chemotherapy [7, 24, 25], gender [17], further extracranial metastases [3, 15, 23] or timing of diagnosis [17] had no influence on the overall survival in our study.

There are two patients in our collective with long-term survival over twelve months; a significant difference compared to the other patients was not verifiable due to the small number. A pronounced diversity is shown in the descriptive analysis regarding age and therapy history. Further investigations with a larger collective are necessary to make a group description for patients with long-term survival.

### Limitations

This study has potential limitations. Due to the retrospective design, a bias cannot be excluded. In addition, because of the study design, only patients who were scheduled for advanced surgical care were included. This selection bias must be taken into account. Furthermore, the sample size was relatively small (*n* = 25). The treatment strategies and the detectability of BM changed due to improved detection methods during the study period of 8 years. In addition, there is a lack of histopathological markers, such as CEA and KRAS, which have been shown to have an influence on survival [16, 26].

### Outlook

It is necessary in a further analysis to extend our results to a larger collective. It is particularly necessary to have a close look at histopathological or even genetic markers in order to make a prognosis estimation depending on the tumor characteristics. This poses a certain challenge since BM in patients with CRC are very rare.

## Conclusion

In summary, we were able to confirm previous characteristics from other studies. We verified the low survival time of 4.9 months particularly regarding overall survival. We identified brain radiation therapy, radiation modality, clinical (KPS) and neurological (MRC-NPS) postoperative performance score in the univariate analysis as important prognostic factors for overall survival. The MRC-NPS is based on the KPS and can be used equivalently. We showed that metastasis volumetrics do not have a significant influence on overall survival in our small cohort.

## Supplementary Information

**Additional file 1: Table S1**. Survival rates after 3 (median survival) and 5 months (mean survival). *P*-value is estimated by log-rank test

**Additional file 2: Table S2**. Univariate survival analysis performed by Cox proportional hazard regression.

## Data Availability

The datasets generated and/or analysed during the current study are not publicly available due ethics approval but are available from the corresponding author on reasonable request.

## Abbreviations

BM: Brain metastasis
CI: Confidence interval
CRC: Colorectal cancer
HR: Hazard ratio
KPS: Karnofsky performance score
MRC-NPS: medical research council neurological performance score
OS: Overall survival
SD: Standard deviation
UICC: Union for International Cancer Control
