# Supplementary material for: The impact of neurological performance and volumetrics on overall survival in brain metastasis in colorectal cancer: a retrospective single-center case series
Source: BMC Cancer. 2022 Mar 28;22:336. doi: 10.1186/s12885-022-09435-1 (PMC8961891; doi:10.1186/s12885-022-09435-1)
Supplement: Supplementary file 1 — Additional file 1: Table S1. Survival rates after 3 (median survival) and 5 months (mean survival). P-value is estimated by log-rank test [file 12885_2022_9435_MOESM1_ESM.docx]

Table S1. Survival rates after 3 (median survival) and 5 months (mean survival). P-value is estimated by log-rank test

|  | | 3-month survival | | 5-month survival | | log-rank test |
| --- | --- | --- | --- | --- | --- | --- |
|  |  | % | ±% | % | ±% | p-value |
| **patient characteristic** | | | | | | |
| gender | female | 66.70 | 19.20 | 50.00 | 20.40 | 0.566 |
|  | male | 84.20 | 8.40 | 84.20 | 8.40 |  |
| age at first diagnosis, yr | > 63 | 69.80 | 14.90 | 58.20 | 16.30 | 0.812 |
|  | ≤ 63 | 40.80 | 13.60 | 40.80 | 13.60 |  |
| high blood pressure | yes | 59.80 | 14.00 | 59.80 | 14.00 | 0.266 |
|  | no | 45.00 | 15.60 | 33.80 | 15.20 |  |
| alcohol consumption | yes | 50.00 | 35.40 | 50.00 | 35.40 | 0.803 |
|  | no | 53.10 | 11.00 | 47.80 | 11.10 |  |
| smoking history | yes | 50.00 | 35.40 | 50.00 | 35.40 | 0.607 |
|  | no | 53.70 | 10.90 | 48.40 | 11.10 |  |
| convulsion | yes | 51.80 | 11.70 | 46.00 | 11.70 | 0.953 |
|  | no | 60.00 | 21.90 | 60.00 | 21.90 |  |
| cardiovascular disease | yes | 66.70 | 19.20 | 50.00 | 20.40 | 0.41 |
|  | no | 48.00 | 12.30 | 48.00 | 12.30 |  |
| diabetes mellitus type II | yes | 33.30 | 27.20 | 33.30 | 27.20 | 0.963 |
|  | no | 55.40 | 11.20 | 49.90 | 11.40 |  |
| second malignoma | yes | 100.00 |  | 100.00 |  | 0.453 |
|  | no | 51.60 | 10.60 | 46.90 | 10.60 |  |
| chronic kidney injury | yes | 37.50 | 28.60 | 37.50 | 28.60 | 0.873 |
|  | no | 55.00 | 11.30 | 49.50 | 11.40 |  |
| chronic obstructive pulmonic disease | yes | 75.00 | 21.70 | 50.00 | 25.00 | 0.959 |
|  | no | 47.80 | 11.70 | 47.80 | 11.70 |  |
| cardiac stents | yes | 100.00 |  | 100.00 |  | 0.997 |
|  | no | 50.80 | 10.80 | 45.70 | 10.80 |  |
| adipositas | yes | 75.00 | 21.70 | 75.00 | 21.70 | 0.407 |
|  | no | 48.40 | 11.60 | 42.40 | 11.60 |  |
| **colorectal cancer** | | | | | | |
| localiyation primarius | colon | 83.30 | 10.80 | 83.30 | 10.80 | 0.315 |
|  | rectum | 92.90 | 6.90 | 69.60 | 12.70 |  |
| UICC stage | n/a | 50.00 | 35.40 | 50.00 | 35.40 | 0.79 |
|  | I | 66.70 | 2720.00 | 66.70 | 27.20 |  |
|  | II | / | / | / | / |  |
|  | III | 46.70 | 16.60 | 46.70 | 16.60 |  |
|  | IV | 46.90 | 18.70 | 31.30 | 17.80 |  |
| UICC stage IV | yes | 85.70 | 9.40 | 62.50 | 17.00 | 0.358 |
|  | no | 87.50 | 11.70 | 70.10 | 1.26 |  |
| Grading | n/a | 62.50 | 21.30 | 20.80 | 18.40 | 0.695 |
|  | G1 | 100.00 |  | 100.00 |  |  |
|  | G2 | 77.80 | 13.90 | 51.90 | 17.60 |  |
|  | G3 | 75.00 | 15.30 | 37.50 | 17.10 |  |
| pulmonary metastasis | yes | 85.70 | 13.20 | 34.30 | 1.95 | 0.578 |
|  | no | 89.50 | 7.00 | 89.50 | 7.00 |  |
| time from first diagnosis to pulmonary metastasis, mo | > 25.5 | 62.50 | 17.10 | 62.50 | 17.10 | 0.208 |
|  | ≤ 25.5 | 42.90 | 17.40 | 42.90 | 17.40 |  |
| liver metastasis | yes | 93.30 | 6.40 | 77.80 | 11.40 | 0.509 |
|  | no | 72.70 | 13.40 | 72.70 | 13.40 |  |
| time from first diagnosis to liver metastasis, mo | > 4 | 62.50 | 21.30 | 62.50 | 21.30 | 0.808 |
|  | ≤ 4 | 62.50 | 17.10 | 46.90 | 18.70 |  |
| other systemic metastasis (without brain) | yes | 66.70 | 15.70 | 44.40 | 16.60 | 0.755 |
|  | no | 82.40 | 9.20 | 82.40 | 9.20 |  |
| extracranial metastasis | yes | 91.30 | 5.90 | 91.30 | 5.90 | 0.794 |
|  | no | 33.30 | 27.20 | 33.30 | 27.20 |  |
| number of chemotherapeutic drugs | > 3 | 45.00 | 16.60 | 33.80 | 15.80 | 0.807 |
|  | ≤ 3 | 57.60 | 13.50 | 57.60 | 13.50 |  |
| systemic chemotherapy | without | 100.00 |  | 100.00 |  | 0.952 |
|  | adjuvant | 80.00 | 10.30 | 72.70 | 11.70 |  |
|  | neoadjuvant + adjuvant | 66.70 | 15.70 | 55.60 | 16.60 |  |
| systemic radiation therapy | without | 85.70 | 9.40 | 77.10 | 11.70 | 0.544 |
|  | neoadjuvant | 50.00 | 25.00 | 0.00 | 0.00 |  |
|  | adjuvant | 75.00 | 15.30 | 50.00 | 11.70 |  |
| **brain metastasis** | | | | | | |
| age at diagnosis of BM, yr | > 66 | 69.30 | 15.00 | 57.70 | 16.30 | 0.767 |
|  | ≤ 66 | 41.30 | 13.60 | 41.30 | 13.60 |  |
| time from first diagnosis to BM, mo | > 51 | 44.00 | 15.50 | 44.00 | 15.50 | 0.548 |
|  | ≤ 51 | 60.60 | 13.90 | 51.90 | 14.40 |  |
| BM | singular | 83.90 | 10.40 | 83.90 | 10.40 | 0.177 |
|  | multiple | 84.60 | 10.00 | 67.70 | 13.40 |  |
| number of BMs | 1 | 85.10 | 9.70 | 85.10 | 9.70 | 0.393 |
|  | 2 | 50.00 | 25.00 | 25.00 | 21.70 |  |
|  | 3 | 62.50 | 21.30 | 41.70 | 22.20 |  |
|  | > 3 | 0.00 |  | 0.00 | 0.00 |  |
| Localization of BM | cerebral | 72.90 | 16.50 | 58.30 | 18.60 | 0.343 |
|  | cerebellar | 50.00 | 20.40 | 33.30 | 19.20 |  |
|  | both | 90.90 | 8.70 | 70.70 | 14.30 |  |
| symptoms of BM | incidental finding | 100.00 | 100.00 | 1.00 | 1.00 | 0.423 |
|  | unspecific CNS | 50.00 | 25.00 | 25.00 | 21.70 |  |
|  | specific CNS | 46.10 | 12.40 | 46.10 | 12.40 |  |
| radiation therapy BM | yes | 63.90 | 11.90 | 63.90 | 11.90 | 0.008 |
|  | no | 21.40 | 17.80 | 0.00 | 0.00 |  |
| radiation therapy modality | stereotactic | 81.50 | 11.90 | 81.50 | 11.90 | 0.009 |
|  | whole brain | 33.30 | 19.20 | 33.30 | 19.20 |  |
| radiation dose, Gy | > 36 | 50.00 | 35.40 | 50.00 | 34.50 | 0.476 |
|  | ≤ 36 | 64.30 | 12.80 | 64.30 | 12.80 |  |
| **surgery** | | | | | | |
| localization surgery | supratentory | 78.80 | 13.40 | 67.50 | 15.50 | 0.203 |
|  | infratentory | 35.90 | 13.90 | 35.90 | 13.90 |  |
| cross total resection | yes | 49.00 | 14.80 | 49.00 | 14.80 | 0.83 |
|  | no | 57.10 | 14.80 | 47.60 | 15.10 |  |
| preoperative KPS | 100 | 100.00 |  | 100.00 |  | 0.855 |
|  | 90 | 50.80 | 17.70 | 50.80 | 17.70 |  |
|  | 80 | 57.10 | 18.70 | 42.90 | 18.70 |  |
|  | 70 | 62.50 | 17.10 | 46.90 | 18.70 |  |
| postoperative KPS | 90 | 100.00 |  | 0.00 | 0.00 | 0.008 |
|  | 80 | 50.00 | 25.00 | 50.00 | 25.00 |  |
|  | 70 | 72.70 | 13.40 | 54.50 | 15.00 |  |
|  | 50 | 0.00 | 0.00 | 0.00 | 0.00 |  |
|  | 40 | 0.00 | 0.00 | 0.00 | 0.00 |  |
|  | 20 | 0.00 | 0.00 | 0.00 | 0.00 |  |
|  | 10 | 0.00 | 0.00 | 0.00 | 0.00 |  |
| postoperative KPS | ≥ 70 | 66.20 | 11.30 | 60.20 | 11.80 | 0.006 |
|  | < 70 | 0.00 | 0.00 | 0.00 | 0.00 |  |
| KPS difference | 0 | 88.90 | 10.50 | 77.80 | 13.90 | <0.0001 |
|  | 10 | 41.70 | 22.20 | 20.80 | 18.40 |  |
|  | 20 | 53.30 | 24.80 | 53.30 | 24.80 |  |
|  | 50 | 0.00 | 0.00 | 0.00 | 0.00 |  |
|  | 60 | 0.00 | 0.00 | 0.00 | 0.00 |  |
|  | 70 | 0.00 | 0.00 | 0.00 | 0.00 |  |
| preoperative MRC-NPS | 1 | 35.60 | 19.90 | 17.80 | 16.00 | 0.531 |
|  | 2 | 60.00 | 15.50 | 6000.00 | 15.50 |  |
|  | 3 | 57.10 | 18.70 | 57.10 | 18.70 |  |
| postoperative MRC-NPS | 1 | 43.80 | 22.60 | 21.90 | 19.20 | 0.001 |
|  | 2 | 71.40 | 17.10 | 71.40 | 17.10 |  |
|  | 3 | 80.00 | 17.90 | 80.00 | 17.90 |  |
|  | 4 | 0.00 | 0.00 | 0.00 | 0.00 |  |
|  | 5 | 0.00 | 0.00 | 0.00 | 0.00 |  |
| postoperative MRC-NPS | ≥ 4 | 0.00 | 0.00 | 0.00 | 0.00 | 0.006 |
|  | < 4 | 66.20 | 11.30 | 0.60 | 11.80 |  |
| MRC-NPS difference | 0 | 64.10 | 11.80 | 57.70 | 12.30 | 0.017 |
|  | 1 | 100.00 |  | 100.00 |  |  |
|  | 2 | 0.00 | 0.00 | 0.00 | 0.00 |  |
|  | 3 | 0.00 | 0.00 | 0.00 | 0.00 |  |
| tumor volume of operated BM, cm^3^ | > 11.18 | 46.20 | 13.80 | 38.50 | 13.50 | 0.683 |
|  | ≤ 11.18 | 61.50 | 15.70 | 61.50 | 15.70 |  |
| preoperative tumor load, cm3 | > 14.59 | 38.50 | 13.80 | 30.80 | 12.80 | 0.406 |
|  | ≤ 14.59 | 71.20 | 14.50 | 71.20 | 14.50 |  |
| postoperative tumor load, cm3 | > 0 | 49.00 | 14.80 | 49.00 | 14.80 | 0.83 |
|  | 0 | 57.10 | 14.80 | 47.60 | 15.10 |  |
| difference tumor load pre-/postoperative, cm3 | > 11.18 | 46.20 | 13.80 | 38.50 | 13.50 | 0.683 |
|  | ≤ 11.18 | 61.50 | 15.70 | 61.50 | 15.70 |  |
| tumor volume cerebellar, preoperative, cm3 | > 4.72 | 40.80 | 13.60 | 40.80 | 13.60 | 0.578 |
|  | ≤ 4.72 | 68.60 | 15.10 | 57.10 | 16.40 |  |
| tumor volume cerebellar, postoperative, cm3 | > 0 | 42.90 | 18.70 | 42.90 | 18.70 | 0.754 |
|  | 0 | 57.10 | 12.50 | 50.00 | 12.80 |  |

*BM*, Brain metastasis; *CI*, confidence interval; *CRC*, Colorectal cancer; *CNS*, central nervous system; *G*, Grading; *Gy*, Gray; *HR*, Hazard ratio; *KPS*, Karnofsky performance status; *Mo*, months; *MRC-NPS*, Medical Research Council Neurological Performance Score; *N*, number; OS, Overall survival; *UICC*, Union for International Cancer Control; Yr, years.
