# Supplementary material for: The impact of neurological performance and volumetrics on overall survival in brain metastasis in colorectal cancer: a retrospective single-center case series
Source: BMC Cancer. 2022 Mar 28;22:336. doi: 10.1186/s12885-022-09435-1 (PMC8961891; doi:10.1186/s12885-022-09435-1)
Supplement: Supplementary file 2 — Additional file 2: Table S2. Univariate survival analysis performed by Cox proportional hazard regression. [file 12885_2022_9435_MOESM2_ESM.docx]

Table S2. Univariate survival analysis performed by Cox proportional hazard regression.

|  | | HR (95 % CI) | p-value |
| --- | --- | --- | --- |
| **patient characteristics** | | | |
| gender, female | | 0.90 (0.32–2.56) | 0.848 |
| age at first diagnosis, yr | | 1.01 (0.98–1.05) | 0.512 |
| age at first diagnosis, > 63 yr | | 0.99 (0.39–2.52) | 0.987 |
| body mass index, kg/cm^2^ | | 0.99 (0.88–1.11) | 0.817 |
| high blood pressure | | 0.67 (0.27–1.69) | 0.397 |
| alcohol consumption | | 0.72 (0.10–5.51) | 0.753 |
| smoking history | | 0.56 (0.07–4.26) | 0.574 |
| convulsion | | 0.96 (0.31–2.98) | 0.943 |
| cardiovascular disease | | 0.61 (0.22–1.73) | 0.353 |
| diabetes mellitus type II | | 0.96 (0.27–3.36) | 0.948 |
| second malignoma | | 0.05 (0–13154.36) | 0.629 |
| chronic kidney injury | | 0.83 (0.19–3.67) | 0.8 |
| chronic obstructive pulmonic disease | | 0.96 (0.31–2.97) | 0.94 |
| cardiac stents | | 0.92 (0.12–7.08) | 0.936 |
| adipositas | | 0.79 (0.23– 2.75) | 0.71 |
| **colorectal cancer** | | | |
| localization primarius, rectum | | 0.72 (0.27–1.87) | 0.495 |
| UICC stage | n/a |  | 0.852 |
|  | I | 1.32 (0.26–6.58) | 0.738 |
|  | II | 0.55 (0.11–2.72) | 0.462 |
|  | III | 0.00 | 0.988 |
|  | IV | 0.65 (0.23–1.90) | 0.435 |
| UICC stage IV | | 1.56 (0.574.29) | 0.386 |
| grading | n/a |  | 0.744 |
|  | G1 | 0.64 (0.07–5.65) | 0.688 |
|  | G2 | 1.12 (0.32–3.94) | 0.856 |
|  | G3 | 1.64 (0.50–5.41) | 0.415 |
| pulmonary metastasis | | 0.82 (0.27–2.44) | 0.717 |
| time from first diagnosis to pulmonary metastasis, mo | | 0.99 (0.97–1.01) | 0.254 |
| time from first diagnosis to pulmonary metastasis, >25.5 mo | | 0.42 (0.13–1.38) | 0.154 |
| liver metastasis | | 0.62 (0.22–1.70) | 0.35 |
| time from first diagnosis to liver metastasis, mo | | 1.00 (0.98–1.01) | 0.648 |
| time from first diagnosis to liver metastasis, >4 mo | | 1.16 (0.34–3.90) | 0.816 |
| other systemic metastasis (without brain) | | 0.77 (0.30–1.96) | 0.583 |
| extracranial metastasis | | 0.90 (0.20–3.98) | 0.885 |
| number of chemotherapeutic drugs | | 1.04 (0.83–1.30) | 0.753 |
| number of chemotherapeutic drugs, > 3 | | 0.81 (0.30–2.22) | 0.679 |
| systemic chemotherapy | w/o |  | 0.98 |
|  | adjuvant | 1.20 (0.15–9.59) | 0.862 |
|  | neoadjuvant + adjuvant | 1.24 (0.15–10.27) | 0.841 |
| systemic radiation therapy | without |  | 0.714 |
|  | neoadjuvant | 0.77 (0.20–2.97) | 0.704 |
|  | adjuvant | 0.65 (0.23–1.84) | 0.415 |
| **brain metastasis** | | | |
| age at diagnosis of BM, yr | | 1.01 (0.97–1.05) | 0.626 |
| age at diagnosis of BM, >66 yr | | 1.02 (0.40–2.59) | 0.968 |
| time of first diagnosis to BM, mo | | 0.99 (0.98–1.01) | 0.262 |
| time of first diagnosis to BM, >51 mo | | 0.69 (0.26–1.79) | 0.439 |
| BM, multiple | | 2.34 (0.82–6.64) | 0.11 |
| Number of BM | | 1.28 (0.94–1.76) | 0.123 |
| Number of BM | >1 | 2.49 (0.88–7.03) | 0.084 |
| number of BM | 1 |  | 0.308 |
|  | 2 | 2.78 (0.72–10.83) | 0.14 |
|  | 3 | 1.92 (0.48–7.62) | 0.354 |
|  | >3 | 3.47 (0.68–17.65) | 0.133 |
| localization of BM | cerebral |  | 0.201 |
|  | cerebellar | 1.06 (0.30–3.69) | 0.928 |
|  | both | 2.63 (0.84–8.22) | 0.097 |
| symptoms of BM | incidental finding |  | 0.23 |
|  | unspecific CNS | 5.27 (0.79–35.34) | 0.087 |
|  | sepcific CNS | 2.44 (0.54–11.02) | 0.247 |
| radiation therapy brain | | 0.25 (0.08–0.80) | 0.019 |
| radiation therapy modality, whole brain | | 5.40 (1.27–22.88) | 0.022 |
| radiation dose, Gy | | 1.01 (0.95–1.07) | 0.802 |
| radiation dose, >36 Gy | | 1.78 (0.34–9.24) | 0.494 |
| **Surgery** | | | |
| localization surgery, cerebellar | | 1.62 (0.62–4.21) | 0.323 |
| cross total resection | | 0.97 (0.38–2.45) | 0.948 |
| preoperative KPS | 70 |  | 0.934 |
|  | 80 | 0.87 (0.27–2.74) | 0.806 |
|  | 90 | 0.69 (0.22–2.14) | 0.517 |
|  | 100 | 0.00 | 0.983 |
| postoperative KPS | 10 |  | 0.193 |
|  | 20 | 0.49 (0.04–6.41) | 0.587 |
|  | 40 | 0.19 (0.01–4.20) | 0.29 |
|  | 50 | 0.06 (0–1.44) | 0.083 |
|  | 70 | 0.04 (0–0.62) | 0.021 |
|  | 80 | 0.04 (0–0.69) | 0.026 |
|  | 90 | 0.03 (0–0.48) | 0.014 |
|  | 100 | 0.00 | 0.989 |
| postperative KPS, ≥70 | | 4.25 (1.25–14.45) | 0.021 |
| KPS difference | 0 |  | 0.062 |
|  | 10 | 0.03 (0–0.46) | 0.013 |
|  | 20 | 0.05 (0–0.91) | 0.043 |
|  | 50 | 0.02 (0–0.45) | 0.013 |
|  | 60 | 0.17 (0.01–3.43) | 0.249 |
|  | 70 | 1.00 (0.06–15.99) | 1 |
| preoperative MRC-NPS | 1 |  | 0.725 |
|  | 2 | 0.63 (0.20–2.00) | 0.429 |
|  | 3 | 0.84 (0.25–2.82) | 0.779 |
| postoperative MRC-NPS | 1 |  | 0.034 |
|  | 2 | 0.44 (0.11–1.75) | 0.242 |
|  | 3 | 0.47 (0.12–1.85) | 0.278 |
|  | 4 | 1.47 (0.27–8.15) | 0.659 |
|  | 5 | 9.12 (1.53–54.31) | 0.015 |
| postoperative MRC-NPS, ≥4 | | 4.25 (1.25–14.45) | 0.021 |
| MRC-NPS difference | 0 |  | 0.078 |
|  | 1 | 0.57 (0.07–4.57) | 0.598 |
|  | 2 | 6.81 (1.49–31.06) | 0.013 |
|  | 3 | 2.75 (0.55–13.78) | 0.219 |
| tumor volume of operated BM, cm^3^ | | 1.05 (0.99–1.12) | 0.116 |
| tumor volume of operated BM,  >11.18 cm^3^ | | 1.36 (0.53–3.47) | 0.524 |
| preoperative tumor load, cm^3^ | | 1.06 (1.00–1.13) | 0.068 |
| preoperative tumor load, >14.59 cm^3^ | | 1.66 (0.66–4.19) | 0.283 |
| postoperative tumor load, cm^3^ | | 1.02 (0.95–1.10) | 0.585 |
| postoperative tumor load, >0 cm^3^ | | 1.03 (0.41–2.60) | 0.948 |
| difference tumor load pre-/postoperative, cm^3^ | | 1.06 (0.99–1.13) | 0.095 |
| difference tumor load pre-/postoperative, >11.18 cm^3^ | | 1.36 (0.53–3.47) | 0.524 |
| tumor volume cerebellar, preoperative, cm^3^ | | 1.04 (0.99–1.09) | 0.13 |
| tumor volume cerebellar, preoperative, >4.72 cm^3^ | | 1.45 (0.58–-3.63) | 0.433 |
| tumor volume cerebellar, postoperative, cm^3^ | | 1.07 (0.82–1.40) | 0.612 |
| tumor volume cerebellar, postoperative, >0 cm^3^ | | 1.06 (0.37–2.99) | 0.918 |

*BM*, Brain metastasis; *CI*, confidence interval; *CRC*, Colorectal cancer; *CNS*, central nervous system; *G*, Grading; *Gy*, Gray; *HR*, Hazard ratio; *KPS*, Karnofsky performance status; *Mo*, months; *MRC-NPS*, Medical Research Council Neurological Performance Score; *N*, number; *n/a*, not applicable, OS, Overall survival; *UICC*, Union for International Cancer Control; Yr, years, w/o, without.
